# Identification and recombinant expression of an antimicrobial peptide (cecropin B-like) from soybean pest *Anticarsia gemmatalis*

**DOI:** 10.1590/1678-9199-JVATITD-2020-0127

**Published:** 2021-03-12

**Authors:** Luís Felipe Costa Ramos, João Henrique de Oliveira Rangel, Guilherme Caldas Andrade, Carolina Lixa, Livia Vieira Araujo de Castilho, Fábio César Sousa Nogueira, Anderson S. Pinheiro, Fabio Mendonça Gomes, Cristiane Dinis AnoBom, Rodrigo Volcan Almeida, Danielle Maria Perpétua de Oliveira

**Affiliations:** 1 Department of Biochemistry, Institute of Chemistry, Center of Mathematical and Natural Sciences, Federal University of Rio de Janeiro (UFRJ), Rio de Janeiro, RJ, Brazil.; 2 Alberto Luiz Coimbra Institute of Graduate Studies and Research (COPPE), Federal University of Rio de Janeiro (UFRJ), Rio de Janeiro, RJ, Brazil.; 3 Carlos Chagas Filho Institute of Biophysics, Federal University of Rio de Janeiro (UFRJ), Rio de Janeiro, RJ, Brazil.

**Keywords:** Antimicrobial peptides, Cecropin B, Heterologous expression, Anticarsia gemmatalis, Agricultural pest

## Abstract

**Abstract:**

**Background:**

Insects can be found in numerous diverse environments, being exposed to pathogenic organisms like fungi and bacteria. Once these pathogens cross insect physical barriers, the innate immune system operates through cellular and humoral responses. Antimicrobial peptides are small molecules produced by immune signaling cascades that develop an important and generalist role in insect defenses against a variety of microorganisms. In the present work, a cecropin B-like peptide (AgCecropB) sequence was identified in the velvetbean caterpillar *Anticarsia gemmatalis* and cloned in a bacterial plasmid vector for further heterologous expression and antimicrobial tests.

**Methods:**

AgCecropB sequence (without the signal peptide) was cloned in the plasmid vector pET-M30-MBP and expressed in the *Escherichia coli* BL21(DE3) expression host. Expression was induced with IPTG and a recombinant peptide was purified using two affinity chromatography steps with Histrap column. The purified peptide was submitted to high-resolution mass spectrometry (HRMS) and structural analyses. Antimicrobial tests were performed using gram-positive (*Bacillus thuringiensis*) and gram-negative (*Burkholderia kururiensis* and *E. coli*) bacteria.

**Results:**

AgCecropB was expressed in *E. coli* BL21 (DE3) at 28°C with IPTG 0.5 mM. The recombinant peptide was purified and enriched after purification steps. HRMS confirmed AgCrecropB molecular mass (4.6 kDa) and circular dichroism assay showed α-helix structure in the presence of SDS. AgCrecropB inhibited almost 50% of gram-positive *B. thuringiensis* bacteria growth.

**Conclusions:**

The first cecropin B-like peptide was described in *A. gemmatalis* and a recombinant peptide was expressed using a bacterial platform. Data confirmed tertiary structure as predicted for the cecropin peptide family. AgCecropB was capable to inhibit *B. thuringiensis* growth *in vitro*.

## Background

Insects are one of the largest and most diverse groups of animals, representing about 80 to 90% of the animal biodiversity [1]. As in other animals, insects must cope with immune challenges from parasites and other pathogens during their life cycle [2]. The digestive tract of insects is the first physical and immune barrier against invading microorganisms that are ingested with food [3]. When this barrier is broken, invading microorganisms are exposed to a variety of cellular and humoral processes that support the host defense [4]. In that regard, the production of antimicrobial peptides (AMPs) is one of the key responses in the humoral immune system [5].

AMPs are cationic peptides usually ranging from 20 to 50 amino acid residues [6] that exhibit broad activity against bacteria, fungi, as well as certain parasites and viruses [7-9]. In insects, these peptides are mainly synthesized in the fat body [10,11], hemocytes, and digestive tract [12]. Cecropins are a well-studied class of antimicrobial peptides, with an α helical structure, first described in *Hyalophora cecropia* hemolymph [13,14] and later described in other lepidopterans and dipterans [15-18]. They belong to a large family of cationic AMPs, which is structured the α-helicoidal secondary structure when they encounter microorganism surfaces [19]. These peptides have shown antimicrobial activity against a wide spectrum of bacteria [20-23] and fungi [24]. 

The soybean caterpillar *Anticarsia gemmatalis* Hübner (Lepidoptera: Noctuidae) is the main soybean defoliating pest in Brazil [25] and is found from the United States to Argentina [26]. At present, *A. gemmatalis* biological control in soybean fields is mostly done by *A. gemmatalis* nucleopolyhedrovirus (AgMNPV) and *Bacillus thuringiensis*. However, studies have shown that *A. gemmatalis* can develop resistance to these agents [27, 28]. In this study, we identified a cecropin B-like peptide from the soybean caterpillar *A. gemmatalis*. We expressed and characterized the mature peptide *in vitro,* and analyzed its activity against three species of bacteria. This peptide is probably produced by the humoral immune system after bacterial challenge and it could be related to resistance mechanisms to biological control with pathogens.

## Methods

### Insects

The *A. gemmatalis* colony was maintained as previously described [29]. Briefly, Larvae were reared on artificial diet and maintained under 25 ± 3°C, 70 ± 10% relative humidity, and 14:10 h (light/dark) photoperiod and collected at the 5^th^ instar for manipulation.

### Bacterial strains

Four bacterial strains were used in this work. *Escherichia coli* DH5α was used for plasmid propagation, while *E. coli* BL21 (DE3) was used for heterologous expression. They were kindly provided by Dr. Bianca C. Neves (Institute of Chemistry, UFRJ, Brazil). *Bacillus thuringiensis kurstaki* strain LFB-FIOCRUZ 475, provided by Dr. Leon Rabinovitch (CCGB/IOC/FIOCRUZ, Brazil), and *Burkholderia kururiensis* strain, provided by Dr. Bianca C. Neves (Institute of Chemistry, UFRJ, Brazil) were used in the antimicrobial tests together with the *E. coli* DH5α strain.

### Sequence analysis by bioinformatics

The entire sequence of cecropin B-like peptide was identified through BLASTX alignment search tool (  https://blast.ncbi.nlm.nih.gov/Blast.cgi) against an *A. gemmatalis* midgut transcriptome analysis (this query sequence was deposited in the GenBank database, code MW330381). A multiple sequence alignment using PRALINE (Center for Integrative Bioinformatics VU) (http://www.ibi.vu.nl/programs/pralinewww) was performed to identify possible conserved regions [30]. The SignalP 4.1 Server program (Department of Bio and Health Informatics) (http://www.cbs.dtu.dk/services/SignalP) [31] was used to map the possible cleavage region. The amino acid sequence of the mature peptide was named AgCecropB. Physico-chemical parameters of the AgCecropB sequence, such as theoretical pI and the expected mass, were determined in ExPASy ProtParam ( https://web.expasy.org/protparam/). Phyre2 (Protein Homology / analogY recognition Engine V 2.0) (http://www.sbg.bio.ic.ac.uk/phyre2/html/page.cgi?id=index) was used to search for structural alignments to generate a structural model for AgCecropB. The suggested template was papiliocin c2la2A. All structure comparisons were performed in PyMOL 4.0 (Schrödinger) (https://pymol.org/2/).

### RNA extraction and cloning of AgCecropB

RNA extracts were prepared using pools of eight 5^th^ instar larvae. Specimens were dissected and midgut epithelia were cleaned and used for total RNA extraction with Trizol® (Invitrogen) [32]. Total RNA was quantified using a Qubit® 2.0 Fluorometer (Invitrogen), following the manufacturer’s recommendations. The RNA samples were analyzed by electrophoresis and used to produce cDNA using the SuperScript III First-Strand Kit (Invitrogen) following the manufacturer’s recommendations.

Cloning was performed as previously described [33], with minor modifications. The DNA sequence of AgCecropB was amplified by PCR. The oligonucleotides were designed and analyzed by using the OligoAnalyzer Tool, from Integrated DNA Technologies^TM^ (https://www.idtdna.com/pages). Forward primer (5'- TTC CAT G **GC GCC CGA GCC TAG GTG G** -3') and reverse primer (5'- GCG CTC GAG *TTA* **TTT TCC TAA GGC TTT TGC** -3') had *NcoI* and *XhoI* restriction site sequences respectively inserted (underlined), complementary AgCecropB sequence regions for primers alignment (bold) and a stop codon sequence was inserted before *XhoI* restriction site sequence (in italic). The template amplification was performed under Platinum Taq standard conditions with 57 °C for annealing temperature. NcoI (New England Biolabs, USA) and XhoI (New England Biolabs, USA) restriction enzymes were used for both amplicon and plasmid digestions. The bacterial expression plasmid pET-M30-MBP which encodes an N-terminal 6XHis tag, followed by maltose-binding protein (MBP) (42.5 kDa) and a TEV protease cleavage site (ENLYFQG), was used to clone AgCecropB [34]. Competent *E. coli* BL21 (DE3) cells were transformed with the ligation mixture and selected for antibiotic-resistant colonies on Luria Bertani (LB) agar containing 100 µg/mL kanamycin.

### Heterologous protein expression

A single colony from freshly transformed bacteria was inoculated in 4 mL of Luria Bertani (LB) broth containing 100 µg/mL kanamycin and cultured for 18 h at 37 °C, 200 rpm in an orbital shaker. The culture was transferred to 25 mL of fresh LB broth containing 100 µg/mL kanamycin and grown at 37ºC, 200 rpm until the optical density at 600 nm (O.D.600) reached 0.6. Subsequently, heterologous expression was induced by 0.5 mM isopropyl-(-D-thiogalactopyranoside (IPTG) (Sigma, USA). Bacterial cells were allowed to grow for 16 h at 28 °C at 200 rpm and 1 mL aliquots were collected at different time intervals to follow recombinant protein expression. These aliquots were centrifuged at 8000 xg for 10 min at 4 °C. The culture precipitates were boiled for 20 min in sample loading buffer [1.7 mM Tris-HCl (pH 7.6), 0.005% bromophenol blue, 0.005% xylene cyanol FF, 10% glycerol, 10 mM ethylenediaminetetraacetic acid (EDTA), 10 mM b-mercaptoethanol and protein content was analyzed by 12% SDS PAGE according Laemmli [35] and stained with Coomassie Brilliant Blue [36]. For large scale expression, the 18 h inoculum was transferred to fresh LB broth (1 L) containing 100 µg/mL kanamycin and grown at 37ºC and 200 rpm until O.D.600 reached 0.6. Hisx6MBP-AgCecropB (recombinant protein) expression was induced with 0.5 mM IPTG and grown for an additional 3 h at 28 °C, 200 rpm. After that, the culture was harvested by centrifugation at 8000 xg for 30 min at 4 °C. Culture precipitate was suspended in 50 mM sodium phosphate buffer, 0.5 M NaCl (pH 7.0) (This solution is named as buffer A in all work) containing protease inhibitor cocktail SIGMAFAST^TM^ Protease Inhibitor Cocktail Tablets, EDTA Free (Sigma-Aldrich) and disrupted by sonication (30s on, 59s off; 15 cycles). The mixture was centrifuged in 8.000 xg 4ºC for 30 min and samples were submitted to 12% SDS-PAGE analysis. The soluble fraction was submitted to purification.

### Purification of AgCecropB

Recombinant AgCecropB was purified by two Ni^2+^-affinity chromatography steps using a 5-mL HisTrap HP (GE Healthcare) column attached to an ÄktaPrime Plus purification system (GE Healthcare). The supernatant fraction was injected onto the column, previously equilibrated with buffer A (as described before), and the recombinant protein was eluted using an imidazole gradient with buffer B [50 mM sodium phosphate (pH 7.0), 0.5M NaCl, 1 M imidazole]. Fractions containing Hisx6MBP-AgCecropB were identified and submitted to dialysis to remove imidazole. The Hisx6MBP tag was removed by proteolysis using an in-house produced His6-TEV at 5:1 ratio (w/w protein:TEV) during 96 h at 4 °C. After cleavage, a second Ni^2+^-affinity purification step was performed to remove His6-TEV protease and Hisx6MBP from AgCecropB. This step was performed in the same conditions described above and analyzed by 18% SDS-PAGE. The recombinant peptide was collected directly in the wash step. AgCecropB concentration was determined using Qubit® 2.0 Fluorometer (Invitrogen) following the manufacturer’s instructions.

### High resolution mass spectrometry (HRMS) of AgCecropB

Sample containing AgCecropB was vacuum dried and solubilized in 50% acetonitrile and 10% ammonium acetate and analyzed by direct infusion on the LTQ Orbitrap Velos mass spectrometer (Thermo Scientific). The mass spectrometer was equipped with a nano-electrospray ionization source (n-ESI) and the sample was infused through nano capillaries (PicoTip Emitter, Glass Tip, coating 1P-4P, New Objective). The source parameters were ionization voltage of 2 kV and temperature 200 °C. The acquisition consisted of a complete scan of the ions in high resolution on the Orbitrap analyzer, with a resolution of 60,000 (for m/z 400), and a range of m / z 400-2000. The acquired spectra were evaluated using the Xcalibur 2.1 software (Thermo Scientific).

### AgCecropB structural analysis

The circular dichroism (CD) spectroscopy and intrinsic fluorescence spectroscopy were performed as previously described [37]. For CD assay, spectra were obtained in a Jasco J-715 1505 model spectropolarimeter (Jasco Corp.), using quartz cuvette 0.1 cm, and analyzed in the region of 190 to 260 nm. AgCecropB spectra were obtained as an average of eight reads in 100 nm/min velocity. Experiments were conducted with the anionic detergent sodium dodecyl sulfate (SDS) (Sigma Aldrich®). The peptide was incubated at different SDS concentrations (50 and 100 µM) for 16 h at room temperature. Baselines (only buffers were used) were subtracted from the peptide spectra. The mean residue molar ellipticity at 222 nm, [θ]222, was plotted in function of SDS concentration. To tryptophan intrinsic fluorescence spectroscopy, assays were acquired in a fluorimeter Cary Eclipse (Varian Inc.). Samples were excited to 280 nm and fluorescence emission of 300 to 420 nm was analyzed. Data of AgCecropB was taken at room temperature in the same concentrations of SDS used in CD assays, with 1 μM concentration of protein.

### Antimicrobial tests

Stock cultures of *B. thuringiensis*, *E. coli* (DH5α), and *B. kururiensis* were plated in nutrient agar medium (HiMedia Laboratories) and incubated for 16 h at 37 °C. One colony from each plate was inoculated in 3 mL of nutrient broth medium (HiMedia Laboratories) and grown for 16 h at 37 °C, 200 rpm. A 250 µL aliquot was inoculated in nutrient broth (25 mL) and cultures were grown for 6 h at 37 °C, 200 rpm to ensure that the exponential phase of growth was reached. O.D.600 was measured and cultures were diluted in ultrapure H_2_O to 0.4 (O.D.600) for antimicrobial tests. 

Assays were carried out in 96-well sterile plates as follows: 100 µL of nutrient broth medium, 50 µL of bacterial growth (final O.D.600 = 0.1), and 50 µL of AgCecropB solubilized in buffer A were added to each well (9 µM AgCecropB in the assay). As a negative control of growth inhibition, 50 µL of buffer was used. Control by using 26.3 µM chloramphenicol was tested. Plates were incubated by 20 hours at 37 °C and read at 600 nm in a Spectra Max M2 plate reader (Molecular Devices). Tests were performed in quintuplicates and all statistical analyses were done in GraphPad 7 Prism. 

## Results

### Bioinformatic analysis of AgCecropB

**Figure 1.**
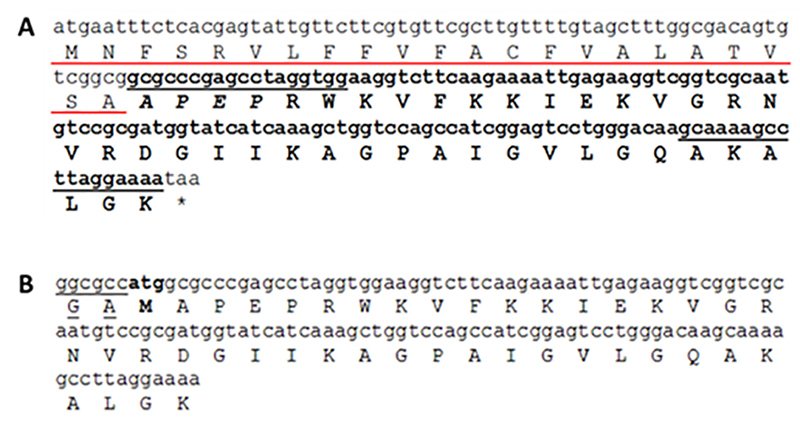
**(A)** Cecropin B-like peptide sequence identified in *A. gemmatalis* midgut transcriptome. Signal peptide sequence (underlined in red) was detected using SignalP software. Underlined nucleotides were used for primer design. Pro region peptide is in italic. **(B)** Recombinant cecropin B-like (AgCecropB) sequence, with remaining residues from TEV cleavage site (underlined in black) and NcoI restriction site (bold).

**Figure 2.**
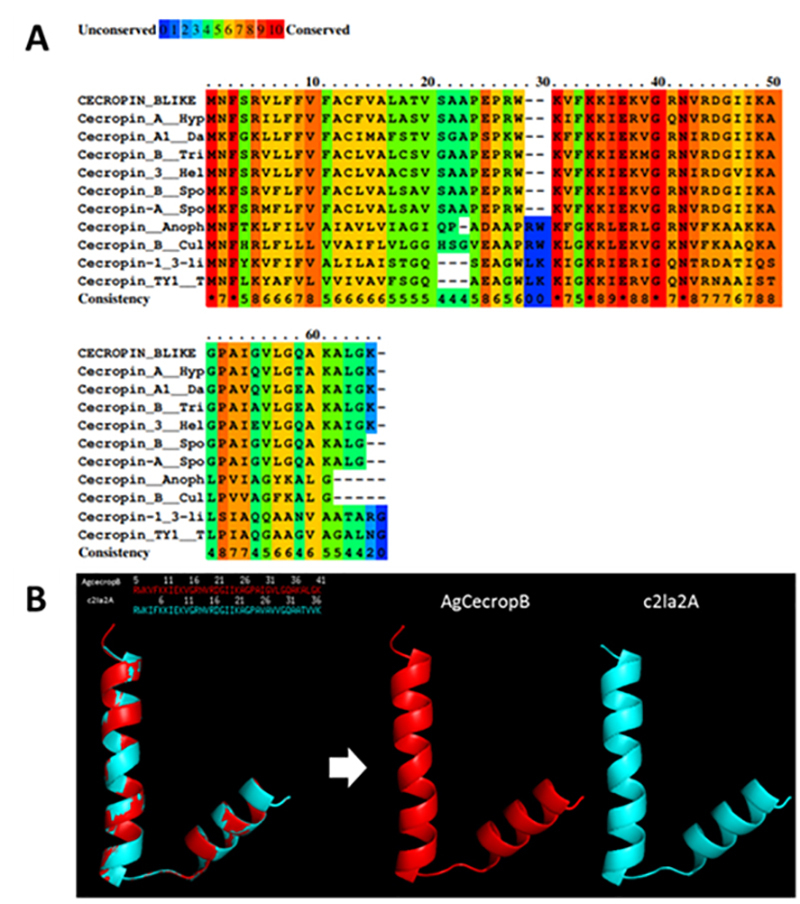
AgCecropB pre-pro-peptide primary sequence alignment and tertiary structure modeling. **(A)** PRALINE alignment result using AgCecropB pre-pro-peptide sequence identified in transcriptome and other lepidoptera and diptera cecropins sequences deposited in GenBank. CECROPIN_BLIKE: Cecropin B pre-pro-peptide from *A. gemmatalis* transcriptome (GenBank database code MW330381); Cecropin_A_Hyp: Cecropin A from *Hyphantria cunea*; Cecropin_A1_Da: Cecropin A1 from *Danaus plexippus plexippus*; Cecropin_B_Tri: Cecropin B from *Trichoplusia ni*; Cecropin_3_Hel: *Helicoverpa armigera* Cecropin 3; Cecropin_B_Spo: Cecropin B from *Spodoptera exigua*; Cecropin-A_Spo: Cecropin A from *Spodoptera litura*; Cecropin_Anoph: *Anopheles darling* cecropin; Cecropin_B_Cul: Cecropin B from *Culex quinquefasciatus*; Cecropin-1_3-li: *Drosophila hydei* 1/3-like cecropin; Cecropin_TY1_T: *Tabanus yao* cecropin 1. **(B)** Overlapping of the tertiary structure models of cecropin B-like peptide and papiliocin c2la2A. Papiliocin c21a2A isolated from *Papilio xuthus* was the suggested template by Phyre2 for AgCecropB tertiary structure modeling. PyMOL generated the overlapping model based on both peptides mature sequence.

The primary sequence of the antimicrobial peptide was identified in an *A. gemmatalis* midgut transcriptome analysis (Figure 1A). This peptide (AgCecropB) has a total of 63 amino acids, with major number of conserved amino acids between positions 31 and 54, which belongs to the mature peptide as observed after PRALINE alignment using other cecropins sequences from Lepidoptera and Diptera order representatives (Figure 2A). Using the *Signal P* 4.1, a cleavage site region was identified between amino acid positions 22 and 23 (two alanine residues) of the complete peptide sequence (data not shown). The theoretical size of the recombinant peptide (4,656.61 Da) and the theoretical pI (10.71) were obtained by ExPASy Tools analysis.

Papiliocin, an antimicrobial peptide that belongs to the cecropin family first described in *Papilio xuthus* [38] was chosen by *Phyre* ^*2*^ as the best template for the modeling of AgCecropB (reference c2la2A). Comparing the suggested structure with AgceCropB, we observed a 90% structure similarity, which showed two α-helixes linked by a hinge, generally formed by a glycine and proline amino acids (Figure 2B).

### Cloning and heterologous expression of AgCecropB

**Figure 3.**
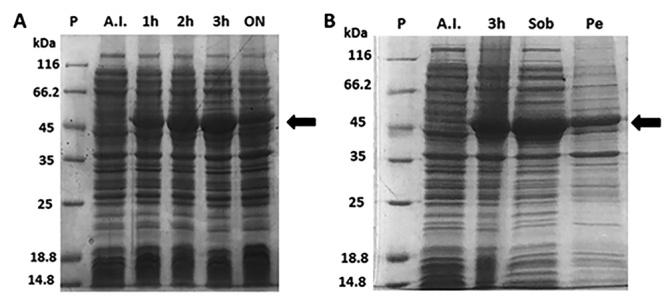
Heterologous expression of recombinant protein Hisx6MBP-AgCecropB and *E. coli* BL21 (DE3) cell lysis. **(A)** 12% SDS-PAGE of Hisx6MBP-AgCecropB expression using IPTG (0.5 mM) to induce expression, at 28 °C. P: molecular mass standard; A.I.: aliquot before induction; 1h, 2h, 3h, 4h, ON: aliquots after 1, 2, 3, 4 and 16 hours after induction. Arrow points to Hisx6MBP-AgCecropB expected molecular mass (≅ 46 kDa). **(B)** *E. coli* BL21 (DE3) cell lysis after 3 hours of induction. P: molecular mass standard; A.I.: aliquot before induction; 3h: 3 hours after induction; Sob: soluble fraction after lysis process; Pe: precipitate fraction after lysis process. Arrow points to Hisx6MBP-AgCecropB expected molecular mass (48.5 kDa).

We designed specific primers for mature peptide amplification. Alanine/methionine residues were added to the recombinant peptide sequence AgCecropB, due to the cleavage site *Nco*I, and a glycine residue remains due to TEV cleavage site (Figure 1B). The partial sequence of AgCecropB was amplified from midgut cDNA pools. The fragment was cloned into a pETM30-MBP plasmid and the sequence was confirmed by colony PCR and sequencing. This cloned sequence was deposited in the GenBank database, code MW310628 and it contains the cloned sequence and predicted protein sequence. *E. coli* BL21 (DE3) cells were transformed for heterologous expression [39] and a protein with the predicted mass (48.5 kDa) of Hisx6MBP-AgCecropB was expressed. Optimal conditions were determined as a 3h induction time using IPTG 0.5 mM (final concentration), at 28 °C (Figure 3A). Following the lysis step, we observed that a large fraction of Hisx6MBP-AgCecropB was found on the soluble fraction (Figure 3B).

### AgCecropB purification

**Figure 4.**
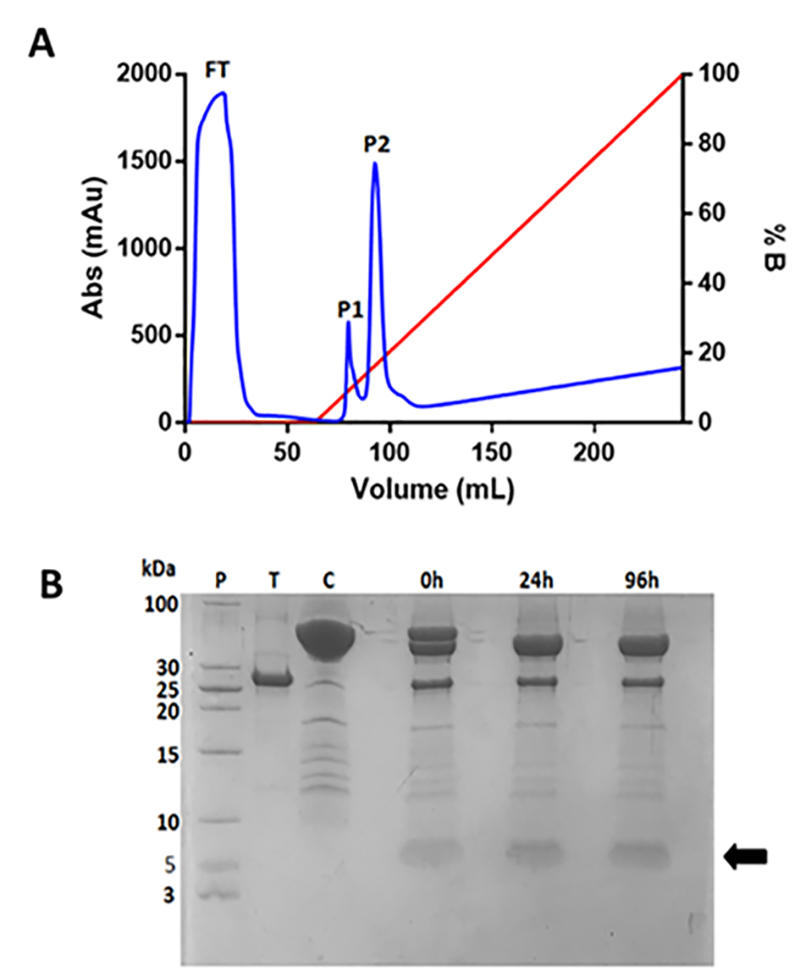
First purification step of recombinant protein and proteolysis assay. **(A)** Chromatogram of Hisx6MBP-AgCecropB purification by affinity Ni^2+^Histrap columns (GE healthcare). Blue line represents sample absorbance measure in 280 nm and red line represent elution buffer B (50 mM sodium phosphate, 0.5 M NaCl, 1 M imidazole [pH 7,0]) concentration during experiment. FT: flowthrough; P1 and P2: protein peaks during chromatography elution. P2 was eluted containing Hisx6MBP-AgCecropB in 160 mM imidazole. **(B)** 18% SDS-PAGE gel of proteolysis assay using TEV endoprotease. P2 fraction was dialysate to extract imidazole and quantified by Qubit for proteolysis assay, using 5:1 proportion (mass/mass) (protein:TEV). P: molecular mass standard; T: TEV endoprotease (28 kDa); C: P2 containing Hisx6MBP-AgCecropB; 0h: aliquot withdrawal at the exact moment TEV and recombinant protein were mixed; 24h and 96h: aliquots of 24 hours and 96 hours, respectively, after cleavage. Arrow indicates recombinant peptide AgCecropB.

**Figure 5.**
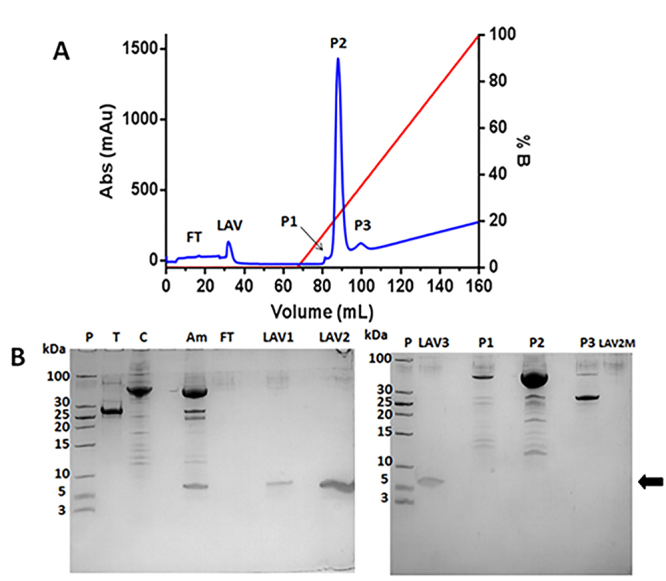
Second purification step to purify AgCecropB. **(A)** Chromatogram purification by affinity Ni^2+^Histrap columns (GE healthcare). Blue line represents sample absorbance measure in 280 nm and red line represent elution buffer B (50 mM sodium phosphate, 0.5 M NaCl, 1 M imidazole [pH 7,0]) concentration during experiment. FT: flowthrough; LAV: peak obtained during washing column buffer (50 mM sodium phosphate, 0.5 M NaCl [pH 7,0]). P1, P2, P3: peaks eluted during buffer B gradient. **(B)** 18% SDS-PAGE gel with chromatography fractions. P: molecular mass standard; T: TEV endoprotease (28 kDa); C: P2 containing Hisx6MBP-AgCecropB; Am: proteolysis sample after 96 hours assay. FT: flowthrough; LAV1, LAV2, LAV3: collected fractions from LAV peak; P1, P2, P3: eluted samples during buffer B gradient; LAV2M: Histrap column sample collected after final washing (2 M imidazole). Arrow indicates recombinant peptide AgCecropB identified in LAV fractions.

Chromatogram of first Histrap Ni^2+^ affinity purification was analyzed, and two peaks were observed (Figure 4A). The recombinant protein was eluted on peak 2 (P2) at 160 mM imidazole concentration. This sample was harvested and the Hisx6MBP tag was cleaved by TEV endoprotease (Fig 4B). The resulting sample was submitted to a new step of affinity chromatography to purify recombinant AgCecropB from Hisx6MBP tag and TEV endoprotease (Figure 5A). One peak containing AgCecropB recombinant peptide was detected during the washing column step before the imidazole gradient (Figure 5B). 

### AgCecropB HRMS and structural assays

**Figure 6.**
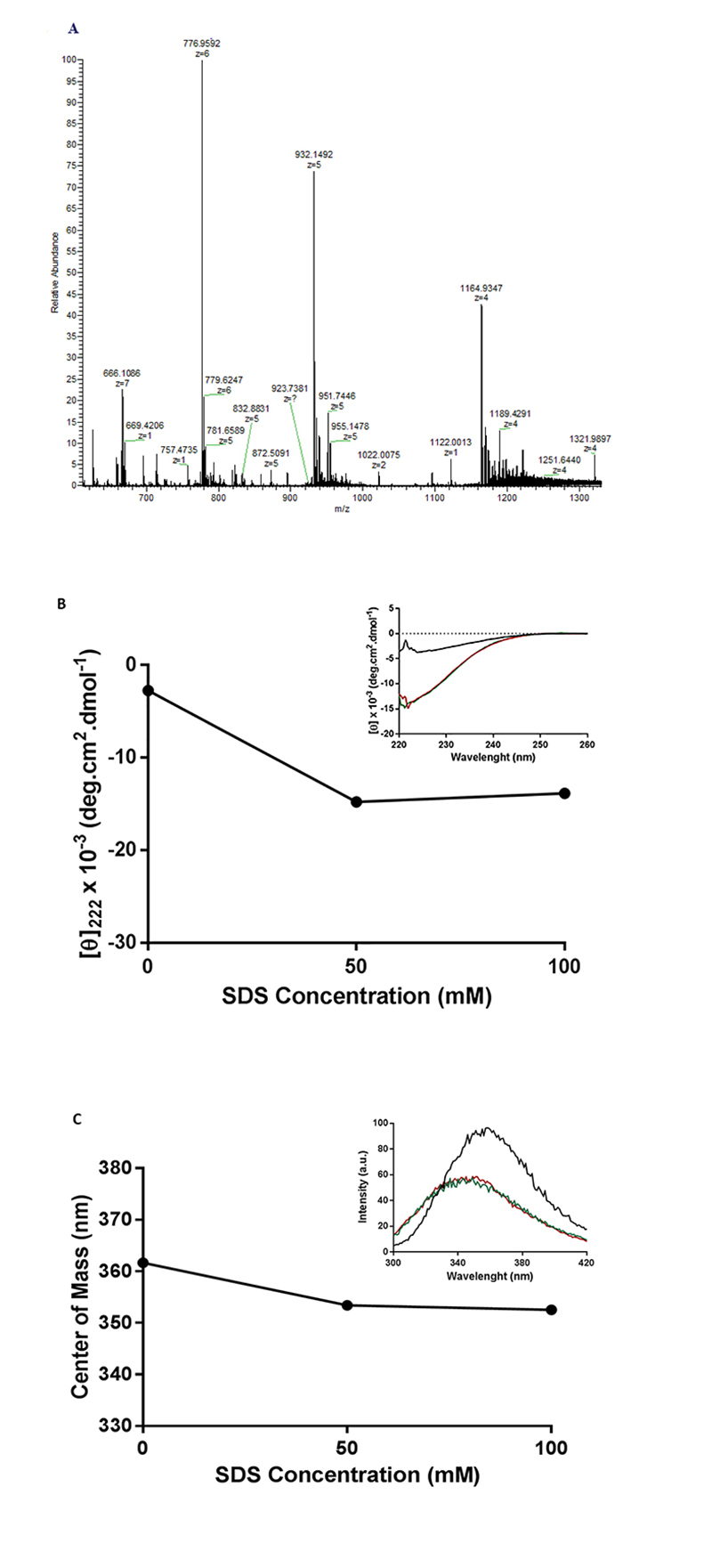
HRMS and secondary structure assays of AgCecropB. **(A)** Mass spectrum chromatogram of purified recombinant cecropin B. On the X axis, the detected fragments and their charge in the spectrum are identified; the Y axis shows the intensity of the fragments, based on their relative abundance. **(B)** Mean residue molar ellipticity at 222 nm of recombinant peptide AgCecropB in the presence of SDS. In the X axis the SDS molar concentration is expressed and in the Y axis is plotted the AgCecropB mean residue molar ellipticity value at 222 nm. Inset: circular dichroism spectrum (220-260 nm). X axis shows the wavelength and in the Y axis the mean residue molar ellipticity. Black line: control without SDS; red line: AgCecropB in the presence of SDS 50 mM; green line: AgCecropB in the presence of SDS 100 mM. **(C)** Center of mass analysis plot at 280 nm. In the X axis the SDS molar concentration is expressed and in the Y axis is plotted the uptake wavelength of AgCecropB center of mass. Inset: tryptophan intrinsic fluorescence assay plot measured at 280 nm; in the X axis is showed the absorption wavelength and in the Y axis the fluorescence intensity. Black line: control without SDS; red line: AgCecropB in the presence of SDS 50 mM; green line: AgCecropB in the presence of SDS 100 mM.

HRMS of recombinant AgCecropB was prepared and the full scan analysis revealed three major peaks: m/z 776.9592 (z = 6), 932.1492 (z = 5), and 1164.9347 (z = 4) (Figure 6A). The obtained average mass is approximately 4,656 Da, confirming the expected mass of recombinant AgCecropB. Structural studies of AgCecropB peptide was performed using circular dichroism and tryptophan intrinsic fluorescence, to obtain information about the secondary and tertiary structure contents of the protein in the presence of a membrane mimicry. CD spectra of the peptide in the presence of two different concentrations (50 and 100 mM) of SDS showed a gain of helical content indicated by the decrease of molar ellipticity value at 222 nm (Fig 6B). The tertiary structural features of the recombinant peptide were monitored by intrinsic fluorescence. The data showed that the spectral center mass decreased in the presence of SDS, with spectrum migration to the red, indicating structural gain (Figure 6C), which corroborates with CD results. It means that the tryptophan, in the presence of SDS, is less exposed to the solvent, probably because of AgCecropB peptide gained structural helical contents or due to its interaction with SDS micelles.

### Antimicrobial tests

**Figure 7.**
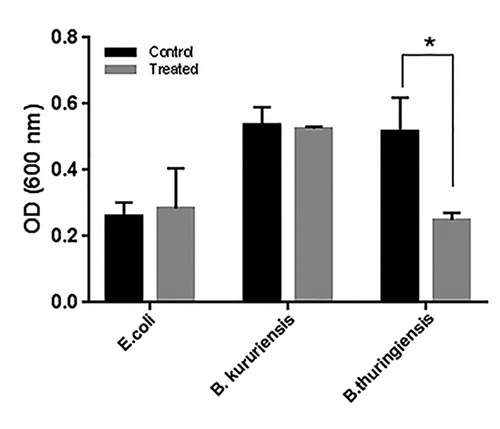
Effect of AgCecropB in the bacterial growth. *E. coli*, *B. kururiensis* and *B. thuringiensis* were exposed to 9 µM of AgCecropB (grey bars) for 20 hours and OD 600 nm was measure. Control samples (black bars) were not exposed to recombinant peptide. Experiment was performed in quintuplicate, and statistical analysis (two-way ANOVA and Bonferroni’s multiple comparisons test) was developed by using the software Graph Pad Prism 7.0 (asterisks mean these compared groups were significantly different).

AgCecropB antimicrobial activity was tested against a gram-positive *(Bacillus thuringiensis)* and two gram-negative (*B. kururiensis* and *E. coli)* bacteria (Figure 7). Chloramphenicol antibiotic inhibition was nearly 90% considering all three bacteria growth, as expected for this antibiotic (positive inhibition control). The data showed AgCecropB inhibited almost 50% of *B. thuringiensis* growth after 20 hours of culture growth. There was no observed effect in *B. kururiensis* or *E. coli*. 

## Discussion

Antimicrobial peptides are important components of the humoral defense of insects, showing activity against gram-positive and gram-negative bacteria, fungi, viruses, and parasites. The action mechanism of most antimicrobial peptides is characterized by the disruption of the microorganism cell membrane [40].

Most cecropin-like peptides have one or two aromatic residues near the N-terminal portion. Tryptophan residues at position 2 and phenylalanine at position 5 are highly conserved on mature peptide [41]. These peptides are generally composed of an amphipathic N-terminal α-helix and a hydrophobic C-terminal α-helix, linked by a hinge region [42,43]. Bioinformatics analysis of cecropin-B-like from *A. gemmatalis* revealed its amino acid sequence homology with other cecropins described in insects, showing that mostly conservative amino acids are comprehended in mature peptide sequence. It was observed by Durell et al. [44] that cecropins N-terminal helix has a net positive formal charge (amphipathic), whereas the C-terminal helix is less charged (hydrophobic), and the hinge between the helices is formed by the glycine and proline residues which are conserved in cecropins, including AgCecropB. We have also predicted tridimensional similarity with papiliocin, a cecropin from the butterfly species *Papilio xuthus,* and the main structural model of cecropins [45,46].

Structural studies of Circular Dichroism and intrinsic fluorescence of tryptophan have been used to analyze the structural features of antimicrobial peptides [41,47,48]. Recombinant cecropin-B-like structural assays suggested an α-helix conformation when AgCecropB is in contact with SDS. This is similar to what has been previously described [49,50]. In an aqueous environment without the detergent, the peptide is found in a random coil structure. In this case, SDS simulates the microorganism membrane, that is negatively polarized due to the presence of the phosphate group in different membrane components. Therefore, this anionic characteristic helps in the action of antimicrobial peptides, which have a cationic charge, resulting in strong interactions. In its natural environment, the peptide only gains structure and activity when in contact with the membrane of a microorganism [51]. As showed in our data, AgcecropB only gained tertiary structure in the presence of SDS, indicating the dependence of a membrane proximity or association to this effect.

To produce the recombinant peptide, a cecropin-B-like partial sequence was cloned in a plasmid containing an MBP solubility tag, a Hisx6 tag, and a cleavage site for TEV endoprotease. Heterologous expression protocols with solubility tags are widely used in the literature [52-55]. These different tags help in the correct folding of protein, solubilization, purification, and protecting the recombinant peptides against the degradation produced by intracellular proteases [56]. MBP tag is a frequently used fusion tag to enhance molecule solubility [57] and is also capable to interact with hydrophobic amino acid residues present in unfolded proteins [58]. In that sense, cecropins have been described as unfolded peptides and the MBP tag could prevent its aggregation. Hisx6-tag is widely used for AMP purification using affinity chromatography steps [59]. MBP and Hisx6 tag were cleaved by TEV endoprotease, that recognizes the short sequence (ENLYFQ↓G) already inserted into the plasmid gene sequence [60]. This protocol is successful and efficient to produce a recombinant and functional peptide, as showed by our data.

Three different bacteria were chosen for antimicrobial assays. AgCecropB did not inhibit the growth of gram-negative bacteria*,* such as *Burkholderia kururiensis* and *E. coli* DH5α. On the other side, AgCecropB was effective against the gram-positive *B. thuringiensis*. This bacteria species is one of the most important biological control agents used against agricultural pests. It produces delta-endotoxins, known as Cry toxins, that have activity against many insects [61], including *A. gemmatalis* caterpillar [62]. Former studies about AMP antimicrobial activity against *B. thuringiensis* showed that AMP encoding genes from *Trichoplusia ni* were increased in insects which were previously exposed to these bacteria [63]. Also, cecropins, moricins and gloverins from *Bombyx mori* showed a high antimicrobial activity against *B. thuringiensis* [64]. In *Spodoptera spp,* the response to B. *thuringiensis* infection depends on several biological factors, such as the production of AMPs [65]. The knockdown of gloverin increased the susceptibility to *B. thuringiensis* in *Spodoptera exigua*, suggesting that this immune response could be related to resistance development [66]. Considering the importance of *B. thuringiensis* for *A. gemmatalis* biological control in soybean fields, it would be worthy to study a possible action of an antimicrobial peptide of this insect in the presence of the bacteria. These analyses could bring some new information about the developing of *A. gemmatalis* resistance to *B. thuringiensis* in the soybean crops.

## Conclusions

A cecropin B-like peptide sequence was identified in *A. gemmatalis* midgut transcriptome and predicted a tertiary structure based on a two α-helix model. AgCecropB was cloned in pETM30-MBP plasmid and expressed using *E. coli* BL21 (DE3). The recombinant peptide was obtained by two steps of affinity purification and a cleavage step using TEV protease. LC-MS/MS and secondary structure data confirmed its molecular mass and the presence of α-helix when exposed to SDS. Antimicrobial tests suggest that AgCecropB can disrupt *Bacillus thuringiensis* growth, which may be a pathway activated by *B. thuringiensis* infection during its use as a biological control for this soybean pest. It would be very interesting to study the correlation between the expression of antimicrobial peptides and the emergence of resistance to Bt.
